# Mr. Hutchins on Galvansim

**Published:** 1803-05-01

**Authors:** William Hutchins

**Affiliations:** 162, Bond Street


### *To the Editors of the Medical and Physical Journal.*

GENTLEMEN,

THE inferences deduced from the late striking experiments in Galvanism, and inserted in your valuable Journal for last month, induce me to take the liberty of offering a few remarks, which, if deemed worthy of insertion, are much at your service.

I am, &c.

162, *Bond Street,*
*April* 10, 1803.

WILLIAM HUTCHINS.

---

ACTUAL experiments, and the facts resulting therefrom, being the only certain means of arriving at a knowledge of the operations of Nature, whether as to their causes, effects, combinations, affinities, or other attendant phenomena, it is of the utmost importance that all deductions drawn from them should be stated, and transmitted to the public with precision and accuracy, whatever theoretical conclusions may arise from investigating and comparing the results: it is from this consideration solely that I presume to trouble you with the following observations, founded on experiments which I have had the honour of attesting, and supported by those I have frequently repeated.

The third inference states, "Not only were the muscles, but the skin and cellular membrane, excited by the Galvanic stimulus." In the experiments alluded to, neither the cellular membrane, cutis, nor moist cuticle, have ever exhibited any evident excitement when the Galvanic arc has been applied to them; they appear to be merely passive conductors, by which property* they convey the Galvanic stimulus to the muscular fibres, in which *alone* excitement is manifested; this is amply corroborated by the experiment of Professor Aldini, where the application of the arc from the spinal marrow to the cellular membrane and cutis, produced powerful contractions in the adjacent muscles.

Under the fourth head it is said, "The contractions of the muscles were excited by the metallic arc, applied to the nerves supplying the muscles; but the nerves themselves were not affected."

Without in the least presuming to appreciate the merit of any set of opinions, may not I be permitted to observe, that late experiments render it doubtful whether the Galvanic impulse is decidedly propagated along the nervous fibres to the muscles they supply, or whether that impulse is conducted merely by the surrounding animal moisture? The experiments of Professor Aldini (several of which were instituted expressly to determine this point) uniformly tend to confirm the latter opinion, particularly experiment twelfth, published in his pamplet, where he observes to his astonishment, that "the Galvanic arc being established from the spinal marrow to the sciatic nerve, divested of its theca, no contraction whatever ensued in the muscles: but the conductors being made to communicate with the fibres of the muscles and cellular membrane, as strong an action as before was manifested." I beg leave here to notice, that in dissecting as much as possible of the theca from the nerve, we also deprive it of a great part of its natural moisture; hence it appears to admit of a question, whether the difference in the action produced in the above experiments is not attributable to the defect or abundance of the animal fluid? Though I shall by no means pretend to decide this question, yet, I feel myself prompted to the suggestion, because I have always found that muscular contractions were excited by the communication of the Galvanic arc, either from nerves to muscles *not* organically united: (so far as anatomy authorises the expression) or from the lungs, cellular membrane, or other moist part, to muscles, or even from one muscle to another, where all organical connection was destroyed by the total excision of both from the body, and nothing but moisture allowed to intervene; and further, because I have never been able to obtain positive results where the circle of communica-

* Dependent perhaps on their lubricating fluid.

tion tion of the Galvanic arc was interrupted by any kind of *dried* animal matter, as bone, nerve, muscle, skin, &c. As most of the experiments demonstrative of the above observations have been publicly displayed in the presence of persons of the most distinguished eminence, they are permanently secured from any imputation that might attach to solitary evidence or premature decision.

Respecting the sixth statement, viz. "That a milky or coagulated matter was formed by repeated contractions of the muscle in contact with the copper wire;"

It is well known, that when water, most animal secreted fluids, saline solutions, &c. are placed in the Galvanic circle, they appear to be decompounded, and a quantity of gas is extricated. In the case before us, upon applying the arc to the muscle, cellular membrane, &c. covered with their natural fluid, or wetted with a solution of muriat of soda, a milky appearance was produced, not from any coagulated matter formed by the contraction of the muscles, for it did not manifest itself till near the conclusion of the series of experiments, when the excitability of the muscles was nearly exhausted, and continued to be generated after all motion had ceased, as long as we continued the application. This phenomenon certainly at first seemed to arise from some peculiar state of the animal matter, induced by the impluse applied; but precisely the same effect occurs, if any of the conducting fluids, as saline solutions, &c. are placed in the circle of communication of the pile, without any animal matter whatever; and the milkiness is caused by the rapid decomposition of the fluid and extrication of gas which rises, forming a white froth or effervesence on the surface.

I come next to consider the seventh and last head, in which it is expressed, that "When the parts ceased to give out motion, the motion was renewed with augmented force, by wetting them with a solution of sal ammoniac."

In Professor Aldini's experiments, as is fairly stated in his Essay, the convulsions of the muscles were increased by the caustic volatile alkali being administered in combination with Galvanism; but the Professor does not mention, nor have I ever known, any instance where the excitability to the Galvanic impulse became in the slightest degree renovated or reproduced (by the application of either caustic, ammonia, or any other known stimulus) after the Galvanic battery of Volta had ceased to excite motion in the muscular fibres; nevertheless, such a stimulus may exist, with which I am however totally unacquainted.

 P. S. Seeing

P. S. Seeing a further account of Professor Aldini's Galvanic experiments in your Journal of the present month, (April) I beg leave to add, that the fluid interposed between the metallic strata in the three troughs was, a *saturated solution of muriat of ammonia*; also, that by mistake of the printer of the Professor's pamphlet, and copied in your work, a considerable misstatement has arisen by the insertion of the word, *no*, instead of, *a very*, in the recital of the elventh experiment, which, according to fact, and agreeable to the manuscript, ought to run thus, " Experiment the eleventh. Conductors being applied from the spinal marrow to the fibres of the *biceps flexor cubiti, glutæus maximus* and the *gastrocnemius*, separately, *a very* considerable action in the muscles of the arm and leg was produced.

